# Association Between Hypothyroidism and Depression in Individuals with Down Syndrome: A Retrospective Analysis

**DOI:** 10.3390/life15081199

**Published:** 2025-07-28

**Authors:** Gregory Sabel, Alishah Ahmadi, Dhruba Podder, Olivia Stala, Rahim Hirani, Mill Etienne

**Affiliations:** 1 School of Medicine, New York Medical College, Valhalla, NY 10595, USA; gsabel@student.nymc.edu (G.S.); aahmadi@student.nymc.edu (A.A.); dpodder@student.nymc.edu (D.P.); rhirani2@student.nymc.edu (R.H.); 2 Department of Neurology, New York Medical College, Valhalla, NY 10595, USA

**Keywords:** down syndrome, depression, hypothyroidism, comorbidities

## Abstract

**Background**: Down syndrome (DS) is a genetic disorder characterized by an extra copy of chromosome 21, often leading to intellectual disabilities, developmental delays, and an increased risk of various comorbidities, including thyroid dysfunction and mental health disorders. The relationship between thyroid dysfunction and mood disorders, particularly depression in DS populations, requires further investigation. **Objective**: This study aims to investigate the presence of a correlative relationship between hypothyroidism and depression in 178,840 individuals with DS, utilizing data from the National Inpatient Sample (NIS) to determine if those with comorbid hypothyroidism exhibit higher rates of depression compared to their counterparts without hypothyroidism. **Methods**: A retrospective analysis of the 2016–2019 NIS dataset was conducted, focusing on patients with DS, hypothyroidism, and depression diagnoses. The diagnoses were determined and labeled based on ICD-10 codes associated with NIS datapoints. Survey-weighted linear regression analyses were employed to assess the association between hypothyroidism and depression within the DS cohort, adjusting for demographic factors such as age, gender, and race. **Results:** This study found that individuals with DS exhibit a significantly higher prevalence of hypothyroidism (29.88%) compared to the general population (10.28%). Additionally, individuals with DS and comorbid hypothyroidism demonstrated a higher prevalence of depression (8.67%) compared to those without hypothyroidism (3.00%). These findings suggest a significant association between hypothyroidism and increased depression risk among individuals with DS. However, the overall prevalence of depression in DS (4.69%) remains substantially lower than in the general population (12.27%). **Conclusions**: This study highlights the importance of considering hypothyroidism as a potential contributor to depression in individuals with DS. Further research is needed to explore the underlying mechanisms of this association and potential screening and management strategies to address thyroid dysfunction and its potential psychiatric implications in DS.

## 1. Introduction

Down syndrome (DS) is a genetic disorder characterized by an extra copy of chromosome 21, affecting approximately 16 per 10,000 live births in the United States [1]. This condition is associated with a wide range of developmental delays, intellectual disabilities, and an increased risk for various comorbidities, including hypothyroidism and mental health disorders.

Previous research demonstrates that individuals with DS have a higher prevalence of thyroid dysfunction compared to the general population [2]. Moreover, newborns with DS have been shown to demonstrate a frequency of congenital hypothyroidism 28 times higher than that of the general population [3]. Thyroid autoimmunity is also common, with studies reporting positive thyroid autoantibodies in 26–39% of DS patients [4,5]. Hypothyroidism, a condition characterized by insufficient thyroid hormone production, is the most common form of thyroid dysfunction observed. Due to the overlap of symptoms between DS and thyroid dysfunction, diagnosis can be challenging, emphasizing the importance of regular thyroid function screening in this population.

Hypothalamic–pituitary–thyroid (HPT) axis dysfunction in Down syndrome is multifactorial, involving both primary thyroidal and central regulatory mechanisms. The most common cause is primary hypothyroidism due to thyroid dysgenesis (e.g., hypoplasia) and autoimmune thyroiditis (Hashimoto’s), both of which are significantly more prevalent in individuals with DS [6,7,8]. Some patients also exhibit abnormal thyroid morphology even in the absence of antibodies, indicating a developmental etiology [7]. Although central hypothyroidism is less common, subtle HPT axis alterations such as elevated TSH with [9] low-normal free T4 have been observed, suggesting possible mild central dysregulation or an altered feedback set-point [10,11].

There are some inconsistencies between previously drawn conclusions regarding the link between psychiatric disorders, particularly depression, and DS. While some studies suggest higher rates of psychiatric morbidity in patients with DS compared to the general population [9,12], other recent evidence does not support an increased prevalence of depression in DS relative to other intellectual disabilities [13]. However, it has been suggested that individuals with DS may have specific vulnerabilities that could increase their risk of developing depression, including neurobiological factors and exposure to stressors [13]. Longitudinal studies indicate that depressive symptoms and social-related problems tend to persist into adulthood for people with DS, while other behavioral issues improve over time [14]. These findings highlight the need for further research on the incidence and risk factors for depression in patients with DS to improve diagnosis and treatment.

The association between thyroid dysfunction and mood disorders, particularly depression, is well-documented in the general population. It has been demonstrated that patients with thyroid dysfunction are more likely to develop depressive symptoms [15]. Hypothyroidism specifically has been associated with an increased risk of depression, particularly in cases of overt hypothyroidism and in female individuals [16]. The relationship involves the hypothalamus–pituitary–thyroid axis, with somatostatin and serotonin playing influential roles [17]. Elevated levels of thyroid-stimulating hormone and thyroid antibodies have been linked to depression and suicide risk [17]. It has even been shown that treating underlying hypothyroidism with thyroid replacement therapy may improve depressive symptoms [17], with T3 administration showing promise in treating antidepressant-resistant depression [18].

Despite these advances, the existing body of research examining the relationship between thyroid dysfunction and depression specifically within the DS population is sparse and inconclusive. Our present study aims to address this paucity by investigating the relationship between hypothyroidism and depression within the DS population using data from the National Inpatient Sample (NIS). Specifically, this study seeks to determine whether individuals with DS who have comorbid hypothyroidism exhibit higher rates of depression diagnosis compared to their counterparts without hypothyroidism. By examining this association, the study aims to inform clinical practice and improve screening protocols for depression in individuals with DS, ultimately contributing to more comprehensive patient care.

## 2. Methods

### 2.1. Database and Patient Selection

This was a retrospective study using data from the 2016–2019 NIS, a nationally representative database of U.S. hospitalizations. Managed by the Healthcare Cost and Utilization Project (HCUP). All data are publicly available through the Healthcare Cost and Utilization Project (HCUP). All diagnostic and procedure codes used for this analysis are available from the authors upon request. Because the data of this analysis reflect publicly available deidentified hospitalization records, it did not meet the requirements for IRB approval.

### 2.2. Study Population

This study focused on three subpopulations within the broader NIS dataset. The subpopulations included individuals with DS, hypothyroidism, and depression, which were tagged with a binary 0 or 1 for the presence of a given diagnosis within a patient’s ICD-10 codes.

Table 1 lists the ICD-10 codes selected for a given diagnosis of interest. Of note, for the depression diagnosis, codes were included for Dysthymic and Cyclothymic disorders due to their similar presentations and overlapping symptoms with other depressive mood disorders. Dysthymia was included due to the DSM-5 relabeling it as persistent depressive disorder under the mood disorder category. For the hypothyroid diagnosis codes, explainable causes of hypothyroidism such as iodine deficiency were excluded, as well as forms of thyroiditis with known etiologies.

### 2.3. Variables and Measures

The primary outcome of interest in this study was the presence or absence of a diagnosis of depression in a patient with DS. These results came in the form of coefficients representing the prevalence of a particular diagnosis. The primary independent variables of interest in this study were the presence of a DS diagnosis, as well as a diagnosis of hypothyroidism as explained above.

### 2.4. Statistical Analysis

Statistical analysis was performed using StataNow 18 BE. To account for the complex survey design of the NIS, we utilized survey-weighted linear regression analyses that incorporated clustering at the hospital level, stratification, and discharge weights. The analysis was conducted using Stata’s survey (svy) commands to ensure proper variance estimation and inference. When examining the relationship between hypothyroidism and depression within the DS population, we employed survey-weighted logistic regression models with year-fixed effects to account for potential temporal trends across the study period (2016–2019). The analysis was also corrected for age at hospitalization. This approach provides more accurate standard errors and population-level estimates compared to traditional statistical tests that assume simple random sampling.

### 2.5. Ethical Considerations

This study utilized the HCUP-NIS, a publicly available database containing de-identified patient information. This secondary analysis of de-identified data was exempt from IRB review under the Common Rule, with data use compliant with HCUP requirements.

## 3. Results

The study population comprised 142,420,378 individuals from the NIS dataset. Demographic characteristics revealed notable differences across the study subgroups (Table 2). In the general population, White individuals represented the majority (62.35%), followed by Black (14.66%) and Hispanic (11.99%) individuals. The DS cohort showed a distinct racial distribution, with a notably higher proportion of Hispanic individuals (20.10%) compared to the general population, while maintaining a similar proportion of White individuals (60.27%). The racial distribution differences between DS and non-DS patients were statistically significant (F = 113.45, *p* < 0.001), with notably higher representation of Hispanic patients in the DS cohort.

Particularly striking was the demographic composition of individuals with concurrent DS, depression, and hypothyroidism, where White individuals comprised 89.92%, with markedly lower representations of Hispanic (3.85%) and Black (2.49%) individuals. Gender distribution also showed notable variations, with females comprising 56.26% of the general population, while the DS population demonstrated a reverse pattern with male predominance (55.32%). Age distribution patterns were markedly different between groups, with the DS cohort showing a younger profile: 50.76% were under 18 years of age, compared to only 14.97% in the general population. Conversely, while 43.14% of the general population was over 60 years old, only 8.68% of the DS cohort fell into this age category, likely reflecting both the younger age of diagnosis and lower life expectancy associated with DS. 

**Figure 1:**
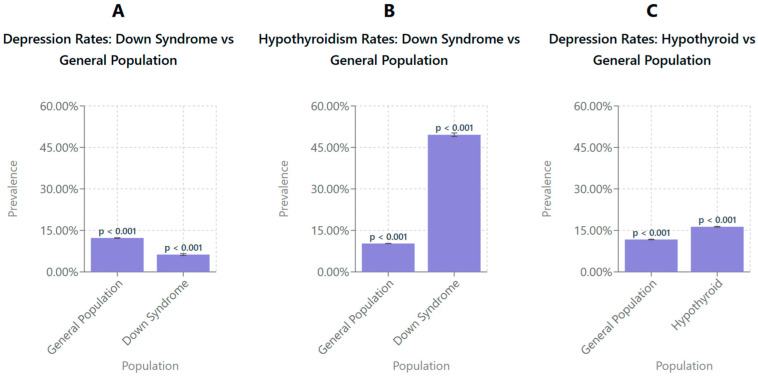
Survey-weighted comparisons of depression and hypothyroidism prevalence between the general population and individuals with DS and between individuals with and without hypothyroidism. (**A**) The prevalence of depression was significantly lower among individuals with DS (6.27%, 95% CI: 5.9–6.6) compared to the general population (12.27%, 95% CI: 12.2–12.4). (**B**) The prevalence of hypothyroidism was significantly higher in individuals with DS (49.57%, 95% CI: 48.9–50.2) than in the general population (10.28%, 95% CI: 10.2–10.3). (**C**) Individuals with hypothyroidism exhibited a higher prevalence of depression (16.32%, 95% CI: 16.2–16.4) compared to the general population (11.72%, 95% CI: 11.6–11.8). All values were estimated using survey-weighted linear regression models adjusted for clustering, stratification, discharge weights, age at hospitalization, and year-fixed effects to account for temporal trends in the National Inpatient Sample (2016–2019). Error bars represent 95% confidence intervals.

Figure 1A shows the prevalence of depression in the general population compared to those with DS. The prevalence of depression in the general population was higher, at 12.27% (95% CI: 12.2–12.4), compared to 6.27% (95% CI: 5.93–6.60) in individuals with DS. These findings suggest a prevalence of depression that is substantially lower among patients with DS relative to the general population. Error bars represent 95% confidence intervals.

Figure 1B shows the prevalence of hypothyroidism in the general population and in the DS population. The findings indicate that the prevalence of hypothyroidism is significantly higher in individuals with DS, 49.57% (95% CI: 48.9–50.2), compared to the general population, 10.27% (95% CI: 10.2–10.3). This suggests that hypothyroidism is more prevalent among individuals with DS relative to the general population. Error bars represent 95% confidence intervals.

Figure 1C shows the prevalence of depression in the general population and patients diagnosed with hypothyroidism. The prevalence of depression was higher in individuals with hypothyroidism, 16.32% (95% CI: 16.2–16.4), compared to the general population, 11.72% (95% CI: 11.6–11.8). This indicates an increased prevalence of depression among individuals with hypothyroidism relative to the general population. Error bars represent 95% confidence intervals.

**Figure 2:**
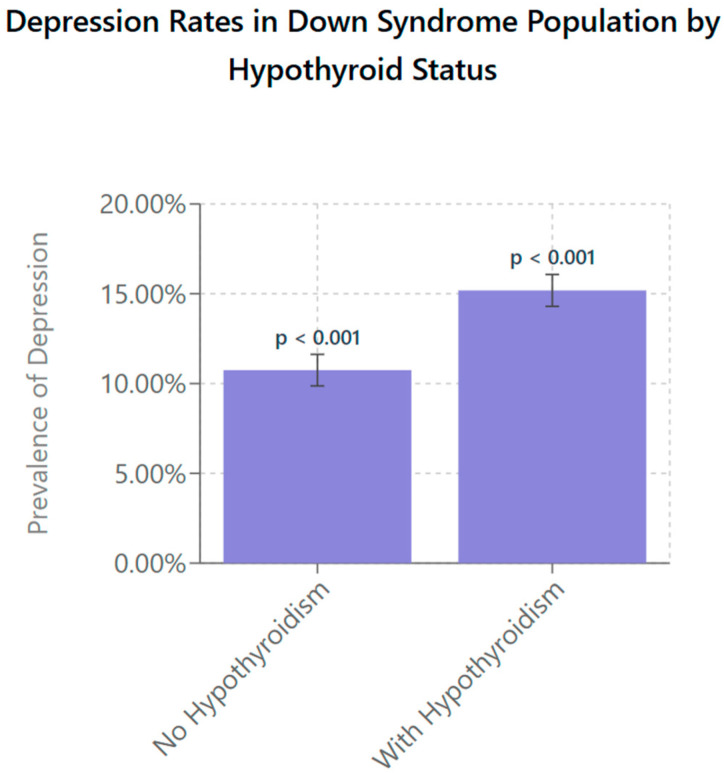
Depression prevalence in individuals with DS stratified by presence of hypothyroidism. Using survey-weighted logistic regression with age and year-fixed effects, individuals with DS and comorbid hypothyroidism were found to have a significantly higher prevalence of depression (15.18%, 95% CI: 14.3–16.1) compared to their euthyroid DS counterparts (10.75%, 95% CI: 9.9–11.6; *p* < 0.001). Models incorporated survey weights, clustering at the hospital level, and stratification to account for the NIS’s complex sampling design. Error bars represent 95% confidence intervals, indicating the precision of the population-level estimates.

Figure 2 shows the prevalence rate of depression in patients with DS with and without hypothyroidism. Patients with DS without hypothyroidism show a depression rate of 10.75% (95% CI: 9.9–11.6; *p* < 0.001), while those with hypothyroidism exhibit a significantly higher rate of 15.18% (95% CI: 14.3–16.1; *p* < 0.001) The error bars represent 95% confidence intervals, highlighting variability within the data and emphasizing the reliability of the observed difference. These findings suggest that the presence of hypothyroidism in individuals with DS is associated with an increased rate of depression.

## 4. Discussion

The primary aim of this study was to examine whether hypothyroidism influences the rate of depression diagnosis in individuals with Down syndrome. We found a statistically significant increase in the prevalence of depression when a hypothyroid diagnosis was also present than if it were not, as seen in Figure 2. Individuals with DS who were diagnosed with hypothyroidism were more than twice as likely as their euthyroid counterparts to have depression with a rate of 8.67% in depression diagnosis compared to 3.00% for their counterparts. This finding presents compelling criteria to utilize for the improvement of screening of depression in DS individuals, as thyroid function tests are already among the most commonly ordered laboratory tests. A study by Ma et al. showed that TSH levels were the third most commonly ordered test in the community in and around Calgary, Alberta, and are among the basic laboratory tests ordered by providers at annual physicals [19].

This relationship between hypothyroidism and depression mimics what is seen in the broad population, as noted by numerous studies including one by Zhou et al. that found a causal relationship genetically between hypothyroidism and Major Depressive Disorder [20]. This schema is reinforced by the current studies data, which showed an increase in the rates of depression of over 7.5% in those with hypothyroidism when compared to their euthyroid peers. While this parallel between DS and general populations might initially suggest redundancy in our findings, the unique medical and social context of DS makes these correlations particularly significant.

Overall, this observation of a higher prevalence of depression in individuals with hypothyroidism compared to the general population warrants a closer examination of the potential interactions between DS, hypothyroidism, and depression. Thyroid dysfunction may exacerbate mood disturbances in patients with DS differently due to the neurodevelopmental and neurochemical changes associated with trisomy 21. Mitochondrial dysfunction, a hallmark of DS, leads to impaired bioenergetics and increased oxidative stress, both of which contribute to neurodegenerative changes and cognitive impairments observed in this population. This dysfunction may exacerbate depressive symptoms by reducing ATP production and impairing neuronal signaling, creating a direct link between cellular energy deficits and mood regulation. These changes could influence how patients with DS respond to thyroid hormone fluctuations, making it critical to understand the underlying mechanisms [21].

### 4.1. Demographic Considerations in Down Syndrome, Depression, and Hypothyroidism

The demographic characteristics of individuals with DS reveal significant disparities in disease prevalence, diagnosis, and outcomes compared to the general population. The over-representation of White individuals (89.92%) with DS, depression, and hypothyroidism, and the underrepresentation of Hispanic (3.85%) and Black (2.49%) individuals, may reflect systemic inequities, such as reduced access to specialized care for minority groups or differences in healthcare-seeking behavior. Diagnostic biases may also contribute to the under recognition of depression and hypothyroidism in these populations. Alexander et al. (2016) reported reduced rates of anxiety and depression diagnoses in individuals with DS, potentially due to difficulties in symptom recognition or diagnostic overshadowing [22].

Gender-specific patterns show a male predominance (55.32%) in the DS population, contrasting with the female majority in the general population. This gender imbalance has implications for screening and treatment strategies. Females with DS may face heightened risks for thyroid dysfunction, while males may experience underdiagnosis of mood disorders due to a lower likelihood of undergoing routine mental health evaluations [23].

Age-related disparities further emphasize the unique healthcare needs of this population. Over half (50.76%) of individuals with DS are under 18 years of age, compared to only 14.97% in the general population, reflecting earlier diagnoses and a reduced life expectancy. This age distribution complicates the interpretation of comorbidities, as younger individuals may not have developed the full spectrum of conditions seen in older age groups. The prevalence of hypothyroidism in children under 3 years with DS is significantly elevated, with an incidence rate ratio (IRR) of 96.3 compared to controls, reinforcing the necessity of early and routine screening from infancy onwards [22,24].

To address these disparities, tailored screening and treatment protocols are essential. Introducing culturally sensitive healthcare practices and addressing language barriers could enhance diagnostic rates and treatment adherence in underserved populations [25]. Employing age-appropriate mental health assessments and emphasizing the importance of routine screenings for thyroid dysfunction and other comorbidities in DS are critical steps [26,27]. These targeted efforts would not only improve diagnostic accuracy but also promote equitable healthcare access and optimize outcomes for all individuals with DS.

### 4.2. The Disparity of Depression Diagnosis in Down Syndrome

The current study found a statistically significant difference in the rate of depression between the general population and those with DS, but in a way that is seemingly counter to previous research. Rivelli et al. (2022) found that individuals with DS have a higher rate of many mental health challenges, specifically among them depression with an odds ratio of 1.27 [1.15,1.39]; *p* < 0.0001) compared to the general population [12]. This is quite different from the results seen in our analysis where the rates of depression were significantly lower in those with DS, at only 4.69% prevalence compared to 12.27% in the general population. One significant factor contributing to the lower recorded prevalence of depression in DS populations may be related to the challenges in diagnosing psychiatric disorders in individuals with intellectual disabilities, especially in the inpatient setting. Studies have noted that depression may manifest differently in this group, with behavioral symptoms often mistaken for other conditions or attributed to intellectual disability itself, leading to under-diagnosis and inadequate treatment [22].

There are several reasons that this disparity may be found between these sets of data: perhaps the most relevant one is a common misconception that individuals with DS are always happy [28], and not appropriately screening these patients for depression in the inpatient setting. This belief is pervasive within the general society and unfortunately also within the medical community and may be a major contributor to the discrepancy. The study run by Rivelli was done in clinics highly familiar with the DS community and specifically tuned to combat stereotypes and preconceived notions about those affected by it.

The clinical settings in which these data are collected may also contribute to disparities. Alexander et al. (2016) noted that general practitioners and primary care providers may be less inclined to diagnose conditions such as depression in individuals with DS, particularly when comorbid conditions like early-onset dementia are present. This highlights the need for specialized mental health evaluations and improved diagnostic criteria tailored to this population [22].

With the context of a potentially widespread misconception about depression prevalence, having effective markers to prompt depression screening in individuals with DS could greatly improve quality of life and mental well-being by those who are overlooked due to their genetic condition. Individuals with DS in particular should have focused monitoring of thyroid levels already, as there is a well-documented predisposition towards thyroid disorders within the group. Given this noted predisposition, we recommend more regular depression screening for DS patients, especially after they have been diagnosed with hypothyroidism. This approach to screening could allow for early identification and intervention for mood disturbances, improving quality of life and combating the progression of depressive symptoms.

### 4.3. Prevalence of Hypothyroidism in Down Syndrome

The present study’s finding of a 29.88% prevalence of hypothyroidism in individuals with DS aligns with the existing literature, which reports rates between 15% and 50%, depending on age and screening rigor. One study found that nearly 50% of individuals with DS experience thyroid dysfunction by adulthood, with about 20% diagnosed before six months of age. Compared to the general population’s prevalence of 10.28%, this nearly threefold increase underscores the need for routine screening [29]. Additionally, congenital hypothyroidism is 28–35 times more prevalent in individuals with DS, likely due to thyroid hypoplasia and reduced follicles, emphasizing the necessity of neonatal screening and early hormone therapy to support neurodevelopment [30].

Further reinforcing this need, Rivelli et al. (2022) reported an odds ratio of 10.94 (95% CI: 10.17–11.78) for hypothyroidism in individuals with DS, highlighting an eleven-fold increased risk compared to controls [12]. Given this disproportionately high prevalence, healthcare plans should prioritize targeted thyroid screening to ensure proper metabolic function and identify those at higher risk for depression [31]. Murphy et al. (2008) demonstrated that capillary TSH measurement via finger prick is a feasible, less invasive alternative to venous sampling, making routine thyroid function monitoring more accessible [32].

These high rates of thyroid dysfunction are driven predominantly by structural and autoimmune thyroid pathology, with additional contributions from HPT axis alterations, immune dysregulation, and oxidative stress [11,33,34,35,36]. Epigenetic modifications, such as aberrant methylation of thyroid-related genes, and factors like selenium deficiency may further impair thyroid function in DS [35,36,37]. Understanding these mechanisms reinforces the importance of rigorous and early screening, as prevention and early treatment of hypothyroidism may mitigate its cognitive and psychiatric sequelae. A comprehensive screening approach can improve quality of life by preventing the cognitive, mood-related, and physical complications of untreated hypothyroidism.

### 4.4. Limitations and Future Research

This study provides valuable insights into the relationship between DS, hypothyroidism, and depression, but several limitations must be acknowledged. The cross-sectional design prevents causal inferences, highlighting the need for longitudinal studies to better understand disease progression and mental health outcomes. Additionally, reliance on inpatient data may introduce bias by excluding outpatients with different clinical profiles, limiting generalizability. The use of ICD-10 codes, while useful for large-scale analysis, may lead to misclassification or variations in diagnostic accuracy. Future research should incorporate outpatient data and standardized clinical assessments to enhance validity. Furthermore, unaccounted confounding factors, such as medication use and social determinants of health, should be controlled to clarify their role in the observed associations.

Future studies could explore whether thyroid hormone replacement, such as levothyroxine, impacts depression rates in individuals with DS, comparing those undergoing treatment to their euthyroid peers. Additionally, the age-related prevalence of hypothyroidism in DS warrants further investigation, as this study found increasing rates across younger age groups, suggesting a need for enhanced screening protocols. Identifying optimal screening intervals could lead to earlier intervention and improved management strategies. Addressing these research gaps will strengthen the evidence base and inform more effective clinical care for individuals with DS.

## 5. Conclusions

Our analysis of the NIS dataset suggests an observed association between hypothyroidism and increased rates of depression within the Down syndrome (DS) population. Individuals with DS who have comorbid hypothyroidism were found to experience higher recorded rates of depression (8.67%) compared to those without thyroid dysfunction (3.00%). While these findings are noteworthy, they should be interpreted cautiously, as the cross-sectional nature of the data limits the ability to infer causality or the directionality of this relationship.

The observed trends carry important clinical implications for the medical management of individuals with DS. They support the importance of regular thyroid function screening in individuals with DS, given the known prevalence of hypothyroidism in this group. Additionally, they highlight the need for enhanced mental health vigilance in DS patients with thyroid dysfunction, which may contribute to improved recognition of underdiagnosed depressive symptoms in this population.

Future research should explore whether thyroid hormone replacement therapy has an impact on mood or depressive symptoms in individuals with DS and further examine the role of age and other comorbidities in moderating this relationship. In conclusion, this study contributes to a growing body of work that underscores the importance of integrated care models for individuals with DS—models that account for both physical and psychological health needs—while also acknowledging the limitations of observational data in establishing definitive associations.

## Figures and Tables

**Table 1:** Diagnosis labels investigated in the current study, as well as the ICD-10 codes used to group patients into the respective diagnostic group.

Diagnosis	Included ICD-10 Codes
Down Syndrome	Q90
Depression	F32, F33, F34.1
Hypothyroidism	E03.0, E03.1, E03.8, E03.9, E06, E06.0, E06.1, E06.5, E06.9

**Table 2:** Demographic Characteristics Across Study Subgroups Including the General Population, Down Syndrome (DS), Depression, Hypothyroidism, and Comorbid DS + Depression + Hypothyroidism.

Characteristic	General Population	Down Syndrome *	Depression *	Hypothyroidism *	DS + Depression + Hypothyroidism *
Total N	142,420,378	178,840	17,455,465	14,664,869	4415
Race/Ethnicity, %					
White	62.35	60.27	76.80	80.76	89.92
Hispanic	11.99	20.10	7.92	7.83	3.85
Black	14.66	11.52	11.26	6.64	2.49
Asian/Pacific Islander	2.99	2.88	1.16	1.90	1.25
Native American	0.64	0.65	0.65	0.50	0.34
Other/Unknown	3.37	4.57	2.22	2.37	2.19
Missing	3.99	-	-	-	-
Sex, %					
Female	56.26	44.68	63.31	73.33	52.48
Male	43.74	55.32	36.69	27.67	47.52
Age Distribution, %					
<18 years	14.97	50.76	3.19	0.55	0.76
18–40 years	21.08	14.44	18.77	7.43	15.55
40–60 years	20.82	26.12	29.50	18.52	64.25
>60 years	43.14	8.68	48.54	73.5	19.44

* These study groups contained statistically different demographic distributions compared to our overall study population.

## Data Availability

Available upon request.
